# Are the Hydantoin-1,3,5-triazine 5-HT_6_R Ligands a Hope to a Find New Procognitive and Anti-Obesity Drug? Considerations Based on Primary In Vivo Assays and ADME-Tox Profile In Vitro

**DOI:** 10.3390/molecules24244472

**Published:** 2019-12-06

**Authors:** Annamaria Lubelska, Gniewomir Latacz, Magdalena Jastrzębska-Więsek, Magdalena Kotańska, Rafał Kurczab, Anna Partyka, Małgorzata Anna Marć, Daria Wilczyńska, Agata Doroz-Płonka, Dorota Łażewska, Anna Wesołowska, Katarzyna Kieć-Kononowicz, Jadwiga Handzlik

**Affiliations:** 1 Department of Technology and Biotechnology of Drugs, Jagiellonian University, Medical College, Medyczna 9, PL 30-688 Cracow, Poland; annamaria.lubelska@doctoral.uj.edu.pl (A.L.); glatacz@cm-uj.krakow.pl (G.L.); marcmalgorzata@gmail.com (M.A.M.); a.doroz-plonka@uj.edu.pl (A.D.-P.); dlazewska@cm-uj.krakow.pl (D.Ł.); mfkonono@cyf-kr.edu.pl (K.K.-K.); 2 Department of Clinical Pharmacy, Jagiellonian University, Medical College, Medyczna 9, PL 30-688 Cracow, Poland; mj.wiesek@gmail.com (M.J.-W.); annairena.partyka@uj.edu.pl (A.P.); daria.wilczynska@doctoral.uj.edu.pl (D.W.); awesolowska@cm-uj.krakow.pl (A.W.); 3 Department of Pharmacodynamics, Jagiellonian University, Medical College, Medyczna 9, PL 30-688 Cracow, Poland; magda.dudek@uj.edu.pl; 4 Department of Medicinal Chemistry Maj Institute of Pharmacology, Polish Academy of Science, Smętna 12, PL 31-343 Cracow, Poland; kurczab@if-pan.krakow.pl

**Keywords:** 5-HT_6_R antagonist, 1,3,5-triazine, hydantoin, ADME-Tox parameters, procognitive effect, obesity

## Abstract

Though the 5-HT_6_ serotonin receptor is an important target giving both agonists and antagonists similar therapeutic potency in the treatment of topic CNS-diseases, no 5-HT_6_R ligand has reached the pharmaceutical market yet due to the too narrow chemical space of the known 5-HT_6_R agents and insufficient “drugability.” Recently, a new group of non-indole and non-sulfone hydantoin-triazine 5-HT_6_R ligands was found, where 3-((4-amino-6-(4-methylpiperazin-1-yl)-1,3,5-triazin-2-yl)methyl)-5-methyl-5-(naphthalen-2-yl)imidazolidine-2,4-dione (KMP-10) was the most active member. This study is focused on wider pharmacological and “druglikeness” characteristics for KMP-10. A computer-aided insight into molecular interactions with 5-HT_6_R has been performed. “Druglikeness” was examined using an eight-test panel in vitro, i.e., a parallel artificial membrane permeability assay (PAMPA), and Caco-2 permeability-, P-glycoprotein (Pgp) affinity-, plasma protein binding-, metabolic stability- and drug–drug interaction-assays, as well as mutagenicity- and HepG2-hepatotoxicity risk tests. Behavioral studies in vivo, i.e., elevated plus-maze (EPM) and novel object recognition (NOR) tests, were performed. Extended studies on the influence of KMP-10 on rats’ metabolism, including biochemical tests, were conducted in vivo. Results indicated significant anxiolytic and precognitive properties, as well as some anti-obesity properties in vivo, and it was found to satisfy the “druglikeness” profile in vitro for KMP-10. The compound seems to be a good lead-structure and candidate for wider pharmacological studies in search for new CNS-drugs acting via 5-HT_6_R.

## 1. Introduction

Serotonin receptors 5-HT_6_ (5-HT_6_Rs) seem to be the most intriguing among the members of 5-HTRs family and also highly promising as a target for innovative therapy of CNS (central nervous system)-diseases. Firstly cloned in rat in 1993 [1,2], and in a human in 1996 [3], they represent the youngest 5-HTRs subtype with a unique distribution that is almost exclusively limited to brain areas, especially those responsible for memory and cognitive processes, i.e., the dorsal hippocampus, striatum and prefrontal cortex (PFC). In the PFC, which is critical to normal cognitive processes, e.g., attention, impulsivity, planning, decision-making, working memory, and the learning or recall of learned memories, the 5-HT_6_Rs occur in pyramidal cells and GABAergic (Gamma-AminoButyric Acid) interneurons, where they regulate neurotransmitter systems that affect the aforementioned processes [4,5]. Thus, the ligands of 5-HT_6_R seem to be pivotal for the successful treatment of cognitive impairment. Predominantly, lines of evidence have indicated memory and learning ability improvement effects, useful in mild and moderate form of Alzheimer’s disease (AD) and age-related cognitive decline as well as antidepressant-like, anxiolytic-like, and anti-obesity properties for 5-HT_6_R antagonists, while similar effects has also been confirmed for some 5-HT_6_R agonists [3,4,6,7,8]. This paradox, although speaking in favor to 5-HT_6_R as a target, is still waiting to be explained. Several hypotheses have postulated the following reasons: (i) the non-selective 5-HT_6_R action of reference ligands used in pharmacologic assays, (ii) the regional and/or (iii) functional specificity of ligands. Each of them is distinctly ligand’s structure-dependent. In this context, a search for new and structurally different ligands for the 5-HT_6_ receptor is in a great scientific importance. 

**Figure 1:**
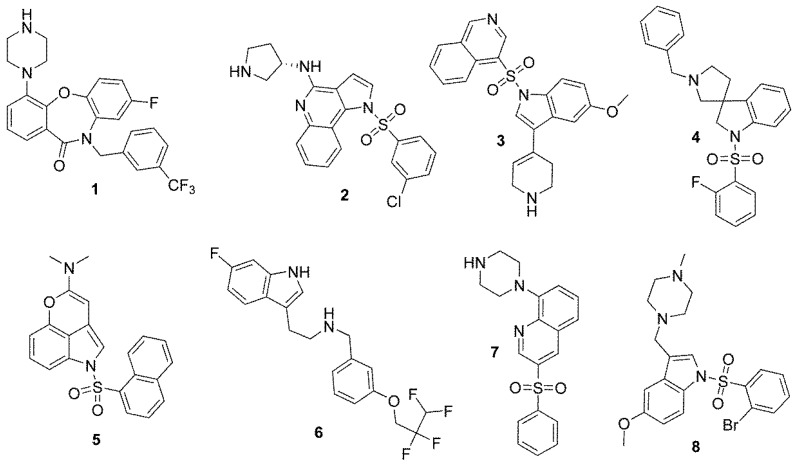
Predominant sulfone- and indole-containing structures of serotonin receptors 5-HT_6_ (5-HT_6_R) ligands found previously: the only one non-sulfonyl and non-indole structure in clinical trials (**1**) [4]; the most active 5-HT_6_R agents that represent the chemical families recently found in primary screenings, i.e., 1*H*-pyrrolo[3,2-c]quinolones (**2**) [11], *N*1-azinylsulfonyl-1*H*-indoles (**3**) [12], spiro[pyrrolidine-3,3′-oxindoles] (**4**) [13] and tricyclic pyrano[2,3,4-cd]indoles (**5**) [14]; the most advanced compounds in clinical trials towards Alzheimer’s disease (AD), i.e., Idalopirdine (**6**), Intepirdine (**7**) and SUVN-502 (**8**) [15].

The results of studies in years 1995–2015 have provided more than 3000 active 5-HT_6_R ligands, but their structural diversity is rather poor, including more than 80% of sulfone-containing structures and more than 40% of indole-containing ones [9]. Among 17 antagonists that have reached clinical trials, only one non-sulfonyl and non-indole structure (**1**, Figure 1) can be found [4,7,10]. The situation has not been improved during last four years, providing new active compounds among 1*H*-pyrrolo[3,2-c]quinolones (**2**) [11], N1-azinylsulfonyl-1*H*-indoles (**3**) [12], spiro[pyrrolidine-3,3′-oxindoles] (**4**) [13] and tricyclic pyrano[2,3,4-cd]indoles (**5**) [14] (Figure 1), thus indicating a strong need to extend the chemical space of 5-HT_6_R ligands.

Furthermore, the above 20-year intensive search for 5-HT_6_R agents has not provided any compound that reached the pharmaceutical market. Among the most advanced ones investigated in potential usage for AD treatment, Idalopirdine (**6**) and Intepirdine (**7**) failed phase III of clinical trials, while SUVN-502 (**8**) gives a great hope because it has been successful in ongoing phase II trials (Figure 1) [15]. An unsatisfactory “drugability,” the so called “ADMET (absorption, distribution, metabolism, elimination, toxicity) profile,” of the earlier investigated 5-HT_6_R ligands was one of the main reasons which disqualified them in the primary stages of drug R&D [16]. Thus, ADMET screening, including an estimation of: (i) bioavailability, (ii) blood–brain penetration, (iii) clearance, and (iv) the risk of toxic effects, is extremely desirable for the potent 5-HT_6_R agents identified in primary pharmacological studies.

The briefly outlined “state of art” significantly underlines a great need to search for new chemical families of 5-HT_6_R agents with a beneficial ADMET profile that would be useful either to give an insight into the molecular level of the agonist–antagonist paradox or in order to find a new solution for the therapy of current civilization diseases.

**Figure 2:**
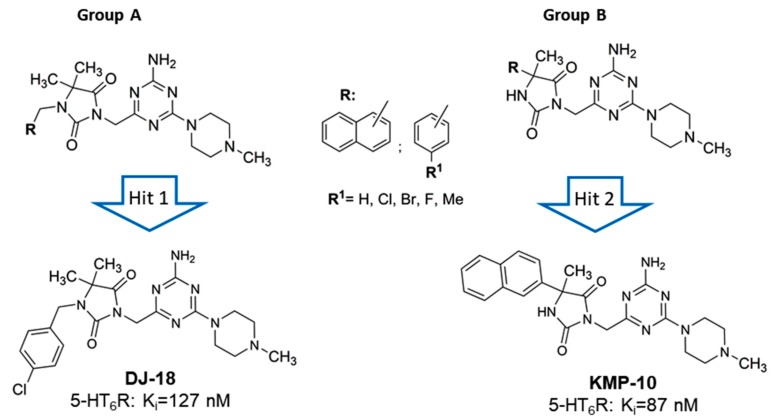
General structures of two series of 1,3,5-triazine hydantoin 5-HT_6_R agents found, Group A and Group B, with respective “hits:” DJ-18 and 3-((4-amino-6-(4-methylpiperazin-1-yl)-1,3,5-triazin-2-yl)methyl)-5-methyl-5-(naphthalen-2-yl)imidazolidine-2,4-dione (KMP-10).

As a response to the aforementioned challenges, we discovered a new chemical class of non-indole and non-sulfonyl 1,3,5-triazine derivatives that displayed a potent affinity and selectivity towards 5-HT_6_R in vitro together with antidepressant-like activity in primary screening in vivo [17,18,19]. Among them, two series that contained the cyclic hydantoin spacer between aromatic moiety and the triazine ring were explored (Figure 2).

The series represent different substitution topologies, i.e., the 5,5-dimethyl-1-arylmethyl-hydantoin derived compounds (Group A) and the 5-aryl-5-methyl-hydantoin derived ones (Group B, Figure 2). Chemical modifications within both groups allowed us to identify two “hits,” DJ-18 and KMP-10, where the last one was a distinctly more potent 5-HT_6_R agent (Figure 2; Table 1) that also showed anxiolytic-like and antidepressant-like activity in vivo. Both compounds displayed anti-obsessive properties in animals fed with the palatable feed and did not show cytotoxic effects on human embryonic kidney (HEK)-293 cells in vitro [19].

In this context, the present study was focused on the wider pharmacological and “druglikeness” characteristics of KMP-10. An insight into molecular interactions of KMP-10 with a 5-HT_6_R receptor in comparison to those of clinically advanced 5-HT_6_R ligands, Idalopirdine and SUVN-502 that belong to different chemical groups, was performed in the first step. Then, “druglikeness” for the compound was examined using a panel of eight ADMET tests in vitro. Behavioral studies in vivo to determine anxiolytic-like and procognitive effects of KMP-10 were also performed. In the last step, extended studies on the influence of the compound on body mass and rats’ metabolism, including biochemical aspects, were carried out.

## 2. Results and Discussion

### 2.1. Molecular Modeling

**Figure 3:**
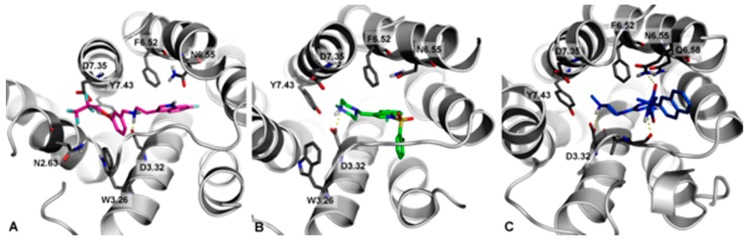
Docking mode to 5-HT_6_R of Idalopirdine (**A**), SUVN-502 (**B**), and KMP-10 (**C**).

Docking studies of KMP-10 in comparison to Idalopirdine (**6**) and SUVN-502 (**8**), representing two different chemical groups of the most advanced 5-HT_6_R antagonists in clinical trials, were performed (Figure 3).

In general, all docked compounds shared a common set of interactions with the 5-HT_6_ receptor, i.e., the salt bridge with D3.32 and CH- π with F6.52. Due to differences in the structure of these ligands (topologies), they showed different binding modes. Idalopirdine (Figure 3A, magenta) represented an extended conformation, where fluoroalkyl chain lied in proximity to asparagine N2.63 (enabling the formation of a hydrogen-bond type of interaction) and aspartic acid D7.35 (in which it can form multipolar orthogonal type of interactions). SUVN-502 (Figure 3B, green) shows a representative binding mode of 5-HT_6_ antagonists [12,17,20], where the 2-Br substituted at the aryl ring was pointed (through the sp3 hybridization of sulfonamide linker) to a hydrophobic cavity formed by helixes 3–5. A slightly different way of interaction from Idalopiridine and SUVN-502 with the 5-HT_6_ receptor showed KMP-10 (Figure 3C, blue). The hydantoin ring formed an additional hydrogen bond with N6.55, which resulted in the anchoring of this fragment in place and the turning of the terminal naphthalene ring towards the extracellular part of the receptor.

### 2.2. ADME-Tox Parameters In vitro

#### 2.2.1. Permeability Assays

##### Parallel Artificial Membrane Permeability Assay (PAMPA)

One of the most commonly used screening methods of compound permeability is the testing of their passive penetration through the bilayer artificial membranes. One of these methods, the pre-coated parallel artificial membrane permeability assay (PAMPA) Plate System Gentest^TM^ (Corning, Tewksbury, MA, USA) [21] was used during this study. This membrane consists of special structure of the lipids and oil that may imitate barriers for compound absorption from the intestines with a good correlation to in vivo conditions. Both tested ligands showed a higher permeability coefficient (*Pe*) than the recommended by the PAMPA Plate System manufacturer value for permeable compounds, which was 1.5 × 10^−6^ cm/s [21], which was much higher than *Pe* estimated during this study for low-permeable reference norfloxacin (Table 2). However, the obtained values differed between the tested 5-HT_6_ ligands. Compound DJ-18, with its *p*-chlorobenzyl substituent, had an excellent, ~1.65 stronger ability to penetrate the artificial membrane than high-permeable reference caffeine, whereas KMP-10, with a naphthyl substituent, showed a ~4-fold weaker permeability than caffeine (Table 2). The result for DJ-18 confirmed our previous data, where the triazine-based 5-HT_6_R ligand with the presence of chlorobenzyl moiety (Compound 1 in the reference [22]) was also shown as highly permeable compound in PAMPA (*Pe* = 23.6 × 10^−6^ cm/s).

##### Permeability Assay with Using Caco-2 Cells

The absorption of compound KMP-10 was also tested in the cell-based Caco-2 assay, which allows for the estimation of permeability in both the passive and active ways. The calculated for KMP-10 permeability coefficient (*P_app_*) was 6.27 × 10^−6^ cm/s (Table 2). That result indicates KMP-10’s moderate permeability according to proposed in the literature classification bands for the Caco-2 permeability model [23], where *P_app_* < 2 × 10^−6^ cm/s means a low permeability, a *P_app_* value from 2 × 10^−6^ to 20 × 10^−6^ cm/s means moderate permeability, and a *P_app_* value above 20 × 10^−6^ cm/s means a highly permeable compound. In the present assay, caffeine was also used as good permeable compound with its calculated *P_app_* = 22.04 × 10^−6^ cm/s. Interestingly, KMP-10 had 4-fold weaker passive penetration than caffeine in PAMPA, whereas it a showed slightly better result in the Caco-2 passive/active transport model, where its absorption was only 3-fold weaker than caffeine (Table 2). Since the literature sources indicated passive transport of caffeine through biological membranes [24], the estimated better result of KMP-10 absorption in the Caco-2 model may be due to some up-take transporters, which are involved in KMP-10 transport through the Caco-2 cells.

Results of both the PAMPA and Caco-2 assays are in good concordance with previously performed blood–brain barrier permeability simulation using QikProp from Schrödinger Suite software. The in silico prediction also showed a moderate permeability properties for KMP-10, with a QPlogBB parameter of −0.92 that was placed almost exactly in the middle of the range predicted for permeable compounds (−0.3–1.2) [18].

#### 2.2.2. Affinity to P-Glycoprotein

**Figure 4:**
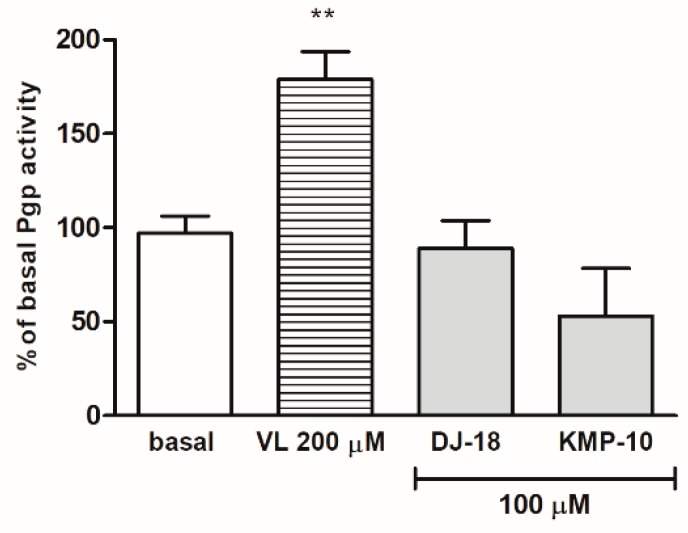
The effect of verapamil (VL) and 5-HT_6_R ligands on P-glycoprotein (Pgp) basal activity (basal). Statistical significance (** *p* < 0.01) was analyzed by Graph Pad Prism^TM^ 6 software using a one-way ANOVA and Bonferroni’s multiple comparison post-hoctest. The compounds were examined in triplicate.

One of the most significant modification strategies for brain penetration improvement is reducing P-glycoprotein (Pgp) efflux. Pgp is active efflux transporter in the lipid bilayer cell membranes of the blood–brain barrier and the intestine. In addition to its influence on drugs’ brain penetration abilities, this integral membrane protein also plays many roles in adverse drug–drug interactions (DDIs) and multidrug resistance. Thus, it is very important to determine as early as possible, if potential drug candidate stimulates or inhibits Pgp. The stimulation of ATP consuming by Pgp is caused by compounds which are substrates for Pgp and may be measured luminescently by Pgp-ATPase (AdenosineTriPhosphatase) assay test. In this assay, the influence of 5-HT_6_ ligands on Pgp basal activity was estimated. The basal activity of Pgp was considered as the difference in the luminescent signal between samples treated with 100 μM of the potent and selective Pgp inhibitor (Na_3_VO_4_) and untreated samples (Figure 4). The stimulation effect was shown for the reference drug verapamil with an increase in ATP consuming up to 175% of Pgp basal activity in a concentration of 200 µM. The results obtained for 5-HT_6_R ligands indicated that none of the tested compounds was the substrate of Pgp because they did not increase the Pgp activity at the tested concentration 100 µM, nor did they did cause any statistically significant changes of basal Pgp activity. No strong substrate of Pgp among triazine-derived 5-HT_6_R ligands had been found in our previous studies as well [22].

#### 2.2.3. Plasma Protein Binding

Binding to the plasma proteins should be investigated in the early stages of the drug discovery process, as this pharmacological effect is exerted only by the unbound fraction (f_u_) of the drug, which can penetrate the cell membranes. Many drugs, especially lipophilic compounds, bind to circulating plasma proteins, such as human serum albumin (HSA), α_1_-acid glycoprotein (AGP), globulins, and lipoproteins [25], especially the HSA and AGP are proteins, which are mostly responsible for the reduction of exposure to drugs. The immobilized HSA and AGP used in this study was included in the commercial test TRANSIL^XL^ PPB (plasma protein binding) assay. The assay mimicked the plasma physiological conditions, where HSA and AGP existed at the ratio of 24:1. Warfarin was used as a positive control that was highly bound to plasma proteins. The effect of this study ~98,5% fraction of warfarin bound to plasma proteins (f_b_) was estimated and found to be similar to the results from the literature [25,26]. In consequence, only 1.5% of the warfarin in the blood stream provided its therapeutic effect. The tested triazine derivative KMP-10 showed a much better result than warfarin, as its f_b_ was calculated at 84.5% (Table 2). Moreover, the calculated equilibrium dissociation constant (K_D_) for KMP-10 was ~10-fold higher than that for warfarin (112 vs. 9.50 µM, respectively; Table 2).

#### 2.2.4. Metabolic Stability

**Figure 5:**
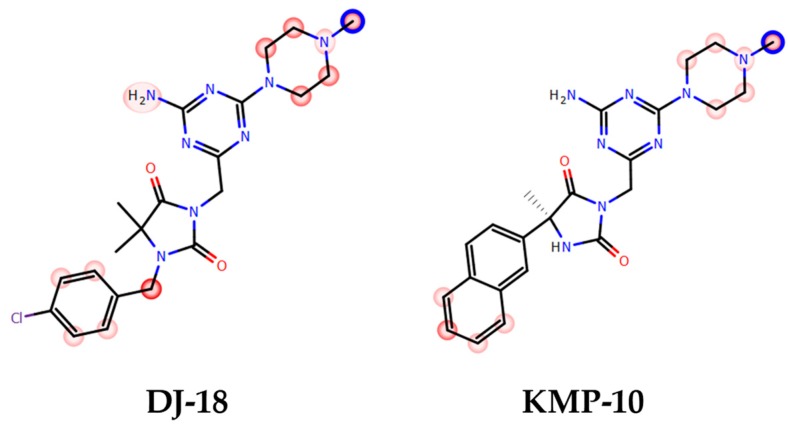
In silico prediction of the sites of metabolism by MetaSite 6.01 for KMP-10 and DJ-18. Blue circle marked on the functional group structures indicates the highest biotransformation probability. The fading red color shows the decreasing of the metabolism probability.

**Figure 6:**
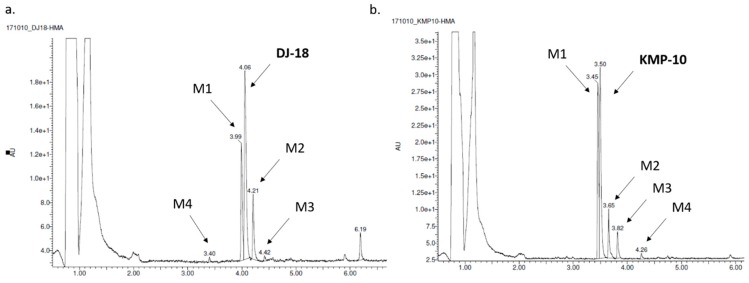
The UPLC spectra of the reaction mixtures after 120 min incubation of DJ-18 (**a**) and KMP-10 (**b**) with human liver microsomes (HLMs).

The most probable structures of metabolites were determined in vitro by HLMs (human liver microsomes) with support of the MetaSite 6.01 software (Figure 5). In silico predictions indicated the same most probable site of metabolism for both compounds, which was *N*-methylpiperazine moiety (Figure 5). Incubation with HLMs for 120 min resulted in the formation of four metabolites by each compound (Table 3; Figure 6a,b). Moreover, the similar main metabolic pathway-demethylation at the *N*-methylpiperazine moiety (the M1 metabolite) was determined for both the 5-HT_6_R ligands by MS and MS/MS analyses (Table 3; Figures S1–S4 in Supplementary Information). That reaction was also predicted by MetaSite with the highest, 100% probability (data not shown) and was observed in our previous investigations on the metabolism of 1,3,5-triazine-methylpiperazine derivatives [22,27]. Other metabolic pathways included hydroxylations (both compounds) and dehydrogenation (KMP-10). The presence of the metabolites that were obtained as the effect of further hydroxylation of the main metabolites was also found (the M3 metabolite of DJ-18 and KMP-10; Table 3).

Apart from the number of metabolites found, the predominant amount of untransformed substrates, either KMP-10 or DJ-18, was confirmed by the analyses, which were able to prove the good metabolic stability of both compounds. The pharmacokinetic properties of DJ-18 and KMP-10 were also determined in vitro using HLMs. Indeed, during the assay, both compounds were found to be metabolically stable and showed similar, low intrinsic clearance *CL_int_* values that were below the value estimated for low clearance compounds *CL_int_* = 8.6 mL min^−1^ kg^−1^ [28] (Table 2). Both compounds also showed a long biological half-life (*t_1/2_*) that was ~6-fold (DJ-18) and ~8-fold (KMP-10) longer than the reference—the unstable drug verapamil (Table 2). The observed good pharmacokinetic properties correspond with results of the previously tested 5-HT_6_R triazine ligands (with a *CL_int_* value also below 8.6 mL min^−1^ kg^−1^) [22]. In this context, the properties of KMP-10 were even better than those of the most active 5-HT_6_R agent found earlier, the thymol-1,3,5-triazine derivative MST4 (4 in the reference [22]), and in the range of the indolemethyl triazine derivative (3 in the reference [22]).

#### 2.2.5. Drug–Drug Interactions (DDIs)

**Figure 7:**
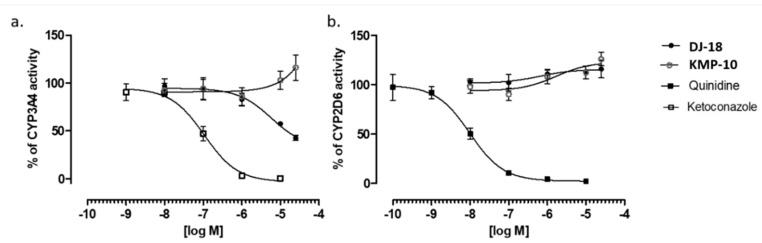
Effect of reference inhibitor (ketoconazole), KMP-10 and DJ-18 on CYP3A4 activity (**a**). Effect of reference inhibitor quinidine, KMP-10 and DJ-18 on CYP2D6 activity (**b**). The compounds were examined in triplicate.

The influence of triazine derivatives on two the most involved in drug metabolism CYP (cytochrome P450) isoforms CYP3A4 and CYP2D6 was also investigated. DJ-18 showed inhibition effects up to ~42% of CYP3A4 activity, but only at the highest concentrations ≥ 10 µM. On the other hand, a slight stimulation effect of CYP3A4 was observed for KMP-10 at the highest used dose of 25 µM (Figure 7a). Both compounds also slightly induced CYP2D6 (Figure 7b). In summary, the compounds showed very low or no risk of DDIs in comparison to the reference inhibitors quinidine and ketoconazole, a results that correlates with previous data obtained for another 5-HT_6_R ligands from the group of 1,3,5-triazine-methylpiperazines [22].

#### 2.2.6. Ames Test

**Figure 8:**
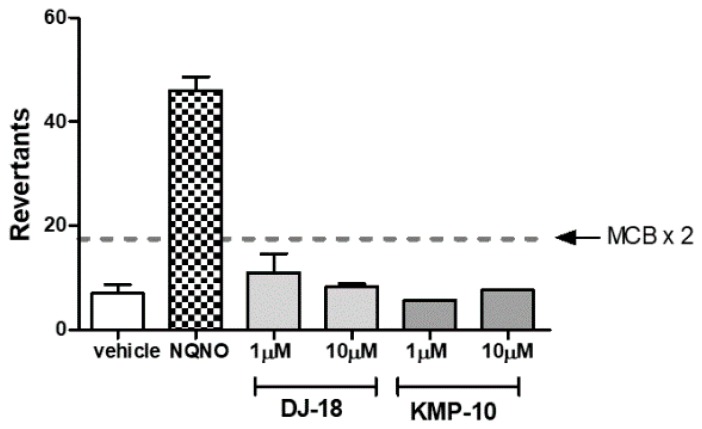
The number of revertants of the *Salmonella typhimurium* TA100 strain exposed to the reference mutagen nonyl-4-hydroxyquinoline-*N*-oxide (NQNO, 0.5 µM) and 5-HT_6_R ligands in two concentrations of 1 and 10 µM. The dashed line marks the doubled medium control baseline (MCB). The compounds were examined in triplicate.

The Ames test received the requirements of OECD (Organisation for Economic Co-operation and Development) and is the “gold standard” for testing of chemicals mutagenicity. The *Salmonella typhimurium* TA100 strain is used in Ames test for detection of mutagens that cause base-pair substitution mutations primarily at one of the GC pairs, which reverted TA100 to the wild-type state. During this study, the medium control baseline (MCB) was calculated first, which is an average of mean revertants (positive wells) from vehicle control (1% DMSO in growth media) plus one standard deviation (SD). Next, it was investigated if the tested compounds were able to cross of the threshold of 2 × MCB, which is considered the mutagen alert. As shown in Figure 8, similarly to our previous results for another triazine-based 5-HT_6_R ligands [22], none of the tested compounds presented any mutagenic effects. The only one which crossed the mutagen alert line was the reference compound nonyl-4-hydroxyquinoline-*N*-oxide (NQNO) at a concentration 0.5 µM. For this reference, 46 positive wells were measured with fold increase over MCB = 5.41 (Figure 8).

#### 2.2.7. Hepatotoxicity Assay

**Figure 9:**
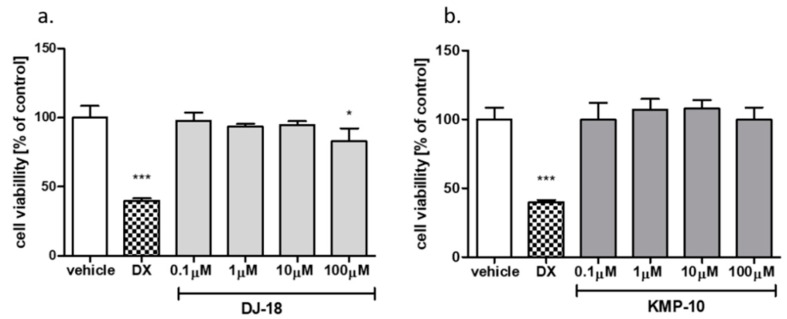
The effect of reference cytostatic drug doxorubicin (DX, 1 µM), DJ-18 (**a**) and KMP-10 (**b**) on the viability of HepG2 cell line. DMSO 1% in cell growth media (vehicle) was used as a control. Statistical significance (*** *p* < 0.001, * *p* < 0.05) was analyzed by Graph Pad Prism^TM^ 6 software using a one-way ANOVA and Bonferroni’s multiple comparison post-hoc test. The compounds were examined in quadruplicate.

The cell-based assays were used to investigate safety of the 5-HT_6_R ligands. In our previous studies [19], the statistically significant slight decrease of HEK-293 cells viability was estimated for DJ-18 at the highest concentration 100 µM. During the same study, no effect for KMP-10 was observed [19]. Additional safety tests with the hepatoma HepG2 cell line were performed here to estimate the compounds’ hepatotoxicity. A slight antiproliferative effect was observed only for DJ-18 at 100 µM, where the cell viability was decreased to up to ~83% of the control (Figure 9a). KMP-10 did not show any hepatotoxic character (Figure 9b). In general, taking into account the results of our present and previous safety assays, all of the to date tested 1,3,5-triazine-methylpiperazines from our compound library showed weak or no cytotoxic activity [19,22].

#### 2.2.8. “Druglikeness” of KMP-10 in Comparison to SUVN-502

In 2017, Nirogi et al. [29] described “druglikeness” profile for SUVN-502 based on both in vitro and in vivo assays. Though the assays were not identical to those we used in our assays presented for KMP-10, they gave some trends of results that can be comparable. Hence, the aforementioned results indicated that SUVN502 is rather strong inhibitor of CYP3A4 and a weaker inhibitor of CYP2D6, while KMP-10 had almost no influence any of the tested CYP isoforms. The pharmacokinetics investigated in vivo for SUVN-502 indicated beneficial values of *t_1/2_* (0.95 h) and *CL_int_* (53 mL/min/kg). The corresponding parameters tested in vitro for KMP-10 were even better (>3 h and 3.74 mL/min/kg, respectively; Table 2). The results of Nirogi et al. demonstrated good BBB (blood brain barrier)-penetration for SUVN-502 after oral administration. Though we did not have the ability to perform the same assays for KMP-10, our in vitro PAMPA and Caco-2 permeability results and suggest an optimistic prognosis for similar behavior in vivo of this hydantoin-triazine derivative. In this context, KMP-10 seems to be promising candidate for further drug R&D.

### 2.3. Behavioral Test In vivo

#### 2.3.1. Anxiolytic-Like Activity of Compound KMP-10

**Figure 10:**
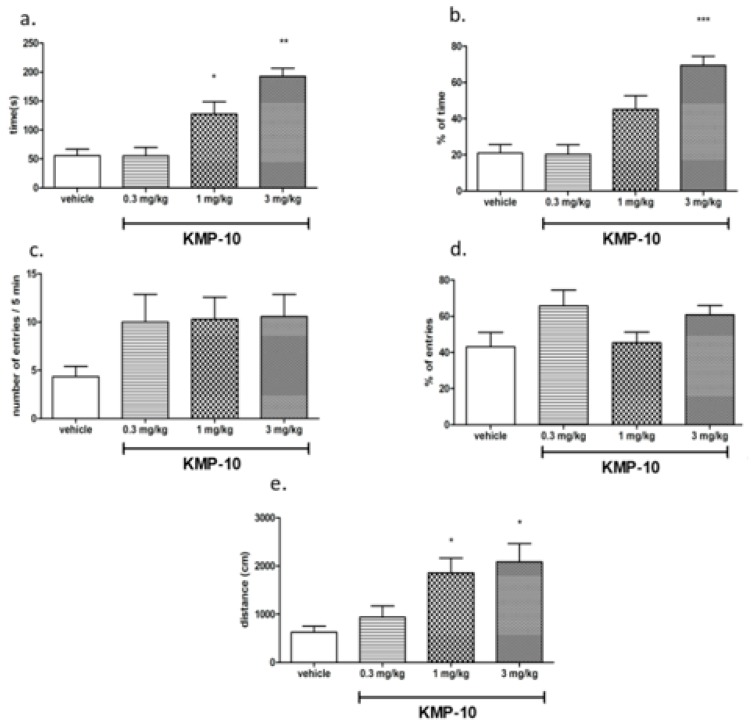
Anxiolytic-like effects of KMP-10 in the elevated plus-maze (EPM) test. Increased open-arm exploration denotes reduced anxiety. KMP-10 was intraperitoneally (i.p.) injected 60 min before the test. Values represent the mean ± SEM of the time (**a**) and percentage of time (**b**) spent in open arms, entries (**c**) and percentage of entries (**d**) distance traveled in the open arms during the five-minute test session (**e**) compared to the respective vehicle group. * *p* < 0.05, ** *p* < 0.01, ***p < 0.001 (ANOVA was followed by the Bonferroni’s post-hoc test), and N = 7–8.

In our previous work [19], the anxiolytic-like effects of KMP-10 were confirmed in a Vogel-conflict test that is the “conditional” anxiety-like assays. This study investigated the anxiolytic-like action in the elevated plus-maze (EPM) test, an “unconditional” assay based on rodents’ natural aversion to heights and open space (Figure 10).

Compound KMP-10 showed anxiolytic-like activity at the doses of 1 and 3 mg/kg. In this test, KMP-10 increased the time spent in the open arms (ANOVA F(3,22) = 17.100; *p* < 0.0001), the percentage of time spent in the open arms (ANOVA F(3,22) = 15.774; *p* < 0.0001) and the distance travelled in the open arms (ANOVA F(3,22) = 5,7922; *p* < 0.01). The observed anxiolytic-like activity of compound KMP-10 was specific, and no changes in locomotor activity were observed in EPM test (data not shown).

#### 2.3.2. Novel Object Recognition (NOR) Test

**Figure 11:**
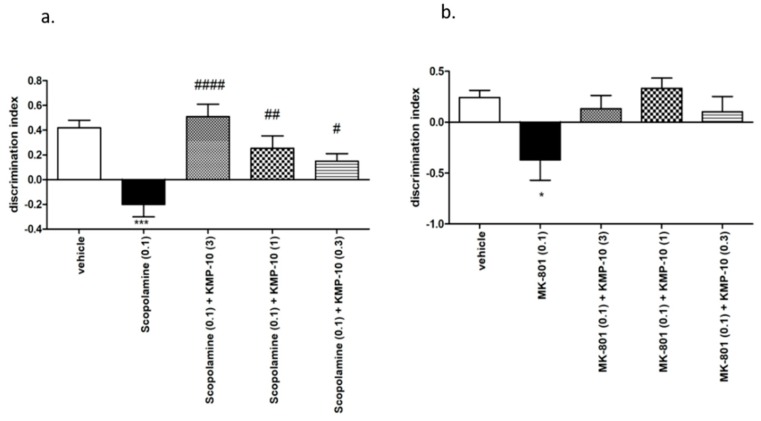
Effect of compound KMP-10 on the memory impairment induced by scopolamine (**a**) and MK-801 (**b**) in the novel object recognition (NOR) test. Compound KMP-10 was i.p. administered 60 min, while scopolamine s.c. and MK were i.p. administered 30 min before the T1 session. The animals were observed for five min. The data are presented as the mean ± SEM of six-to-eight rats. The data were statistically evaluated by a one-way ANOVA followed by Bonferroni’s post-hoc test, * *p* < 0.05, and *** *p* < 0.001 vs. respective vehicle group, and # *p* < 0.05, ## *p* < 0.01 and #### *p* < 0.0001 vs. respective scopolamine treated group. For the one-way ANOVA for compound KMP-10 for scopolamine-induced memory impairment, F(4,31) = 10.640; *p* < 0.0001, and, for the MK-801-induced memory impairment, F(4,29) = 1.000; NS (not significant).

KMP-10 at the full dose-range (0.3–3 mg/kg) significantly and dose-dependently reversed the memory deficits induced by scopolamine in the novel object recognition (NOR) test, while during the memory impairment induced by MK-801, some activity at the dose of 1 mg/kg of KMP-10 was observed, but the discrimination index did not reach a statistically significant level (Figure 11).

### 2.4. Metabolic Test In vivo

In order to extend the previously investigated [19] basic influence of KMP-10 on food-induced the body-mass increase, this study was focused on deeper insight into the action, i.e.,: (i) the influence on caloric and water intakes of either obese or standard fed rats, (ii) the influence of the three-week chronic treatment with KMP-10 on the amount of peritoneal adipose tissue and liver mass, (iii) the influence of KMP-10 on blood glucose, cholesterol and triglyceride levels in the model of excessive eating, and (iv) the influence of KMP-10 on a spontaneous activity test.

#### 2.4.1. Influence of KMP-10 on Caloric and Water Intakes of Obese Rats

**Figure 12:**
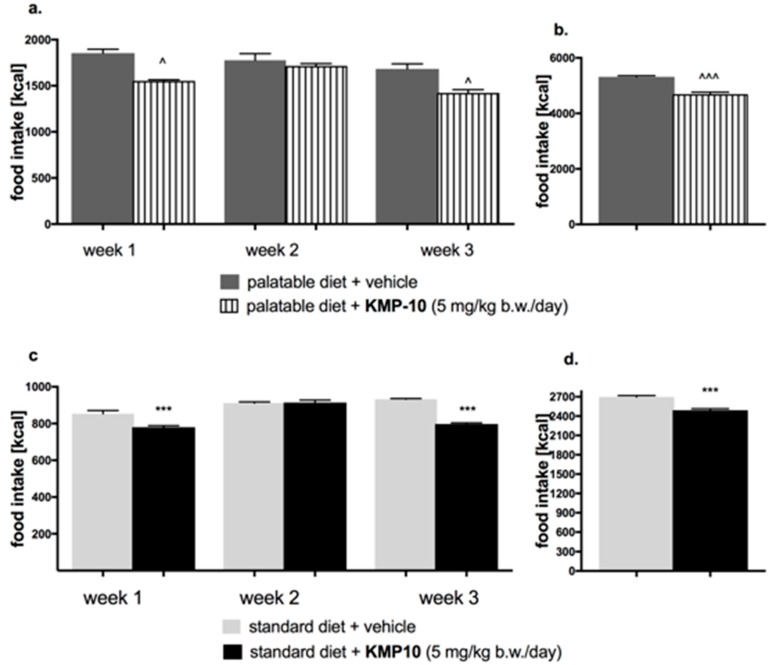
Effect of long-term administration of KMP-10 on food intake in male Wistar rats (**a**). Food intake by male Wistar rats fed with palatable diet in particular weeks (**b**). Sum of food intake by male Wistar rats fed with palatable diet (**c**). Food intake by male Wistar rats fed with standard diet in particular weeks (**d**). Sum food intake by male Wistar rats fed with standard diet. Results are mean data for two animals housed together, n = 8. ^ Comparisons *vs.* the vehicle-treated control group fed palatable diet; * comparisons versus the vehicle-treated control group fed standard diet, the two-way ANOVA (**a**,**c**) and the Student’s *t*-test (**b**,**d**); ^^, ** *p* < 0.01; and ^^^, *** *p* < 0.001.

**Figure 13:**
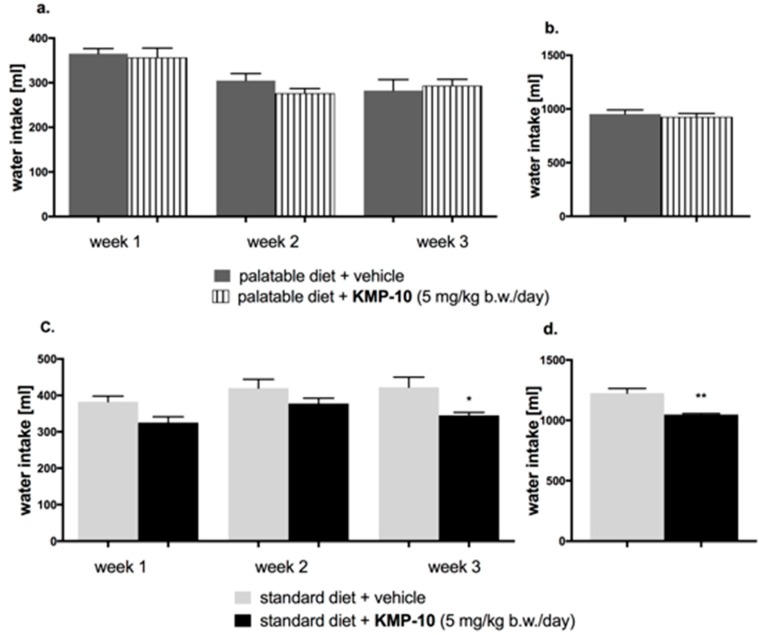
Effect of long-term administration of KMP-10 on water intake in male Wistar rats (**a**). Water intake by male Wistar rats fed with palatable diet in particular weeks (**b**). Sum of water intake by male Wistar rats fed with palatable diet (**c**). Water intake by male Wistar rats fed with standard diet in particular weeks (**d**). Sum of water intake by male Wistar rats fed with standard diet. Results are mean data for two animals housed together, n = 8. * Comparisons versus the vehicle-treated control group fed standard diet, a two-way ANOVA (**a**,**c**) and the Student’s *t*-test (**b**,**d**); * *p* < 0.05 and ** *p* < 0.01.

In the tested group, KMP-10 intraperitoneally (i.p.) administered significantly reduced the amount of calories consumed by the animals (starting from the first week of the experiment), but not water intake, in comparison to the obesity control group (Figure 12a,b and Figure 13a,b).

#### 2.4.2. Effect of KMP-10 on Calorie and Water Intakes in Rats Fed Standard Diet

Starting from the first week of the experiment, KMP-10 i.p. administered at a dose of 5 mg/kg b.w. significantly reduced the amount of calories consumed by the animals in the test group as compared with the control group. Results are shown in Figure 12c,d. A significantly lower water intake in the tested groups as compared to the control group was also observed (Figure 13c,d).

#### 2.4.3. Influence on the Amount of Peritoneal Adipose Tissue and Liver Mass

**Figure 14:**
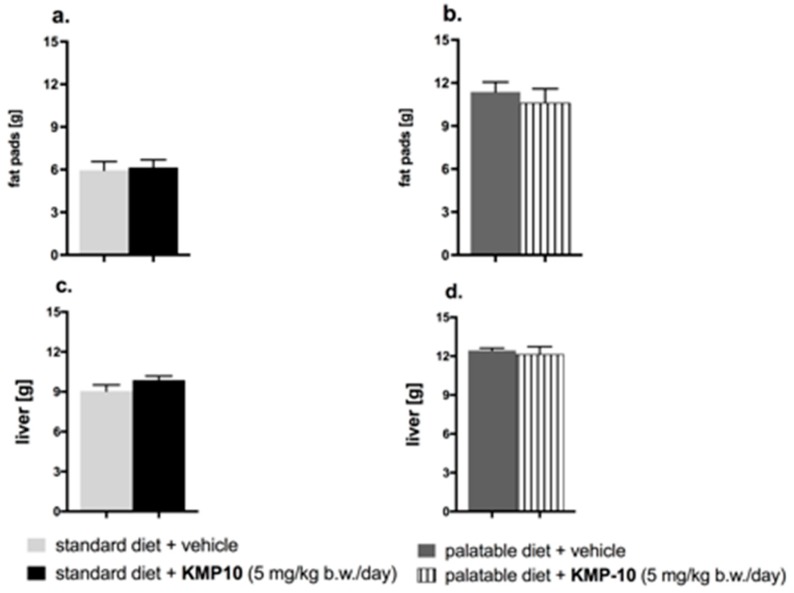
Effects of long-term administration of KMP-10 on amount of peritoneal fat (**a**,**b**) and liver weight (**c**,**d**) in male Wistar rats. Results are means ± SEM, n = 8. Comparisons were performed by a Student’s *t*-test.

The influence of the three-week chronic treatment with KMP-10 on the amount of peritoneal adipose tissue and liver mass is shown in Figure 14.

Animals consuming the palatable feed had a significant increase of fat in peritonea vs. animals consuming the standard feed. The fat pads in the animals from the control group fed the palatable feed weighed, on average, about 5.42 g more than in animals from the control group fed the standard feed (increase by 91.5%) (*** *p* < 0.001; Student’s *t*-test). The group that received the tested compound had a comparable amount of fat in the peritonea, as compared to the control rats (Figure 14a,b). Liver in the animals from control group fed the palatable feed weighed, on average, about 3.38 g more than in animals from the control group fed the standard feed (increase by 37.3%) (*** *p* < 0.001; Student’s *t*-test). The livers in the tested groups had about the same mass as the livers in the control groups (Figure 14c,d).

#### 2.4.4. Influence of Diet or of KMP-10 on Blood Glucose, Cholesterol and Triglyceride Levels in the Model of Excessive Eating

**Figure 15:**
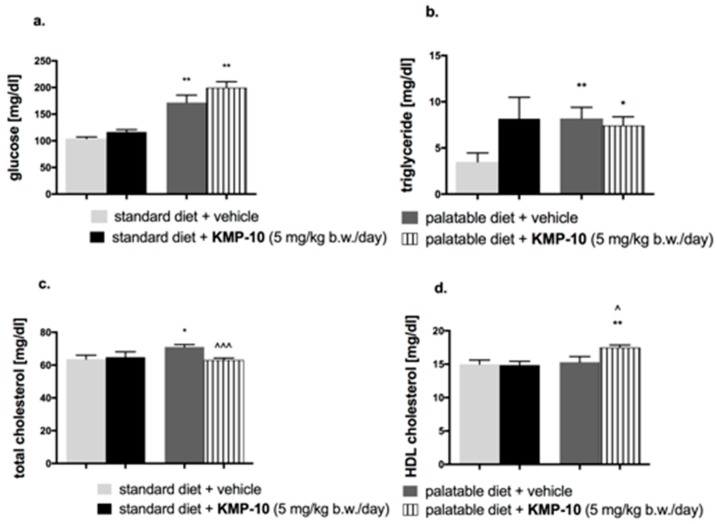
Effects of diet or of long-term administration of KMP-10 on blood glucose level (**a**), blood triglyceride level (**b**), blood total cholesterol level (**c**), or blood HDL (High-density lipoprotein) cholesterol level (**d**) in male Wistar rats in the excessive eating model. Results are means ± SEM, n = 8. Concentrations in blood: mg/dl. Comparisons versus the vehicle-treated control group (*) or versus the vehicle-treated excessive eating control groups (^) were performed by the Student’s *t*-test, significant differences are denoted by *, ^ *p* < 0.05, ** *p* < 0.01, ^^^ *p* < 0.001.

In animals fed with the palatable feed, the blood glucose level and blood triglyceride level were significantly higher than those in the control group fed the standard feed; results are shown in Figure 15a,b. KMP-10, at a dose of 5 mg/kg b.w., significantly decreased the total cholesterol in blood in comparison to the group fed the palatable feed (Figure 15c) and significantly increased HDL (high density lipoprotein) cholesterol (Figure 15d).

#### 2.4.5. The Influence of KMP-10 on Spontaneous Activity Test

**Figure 16:**
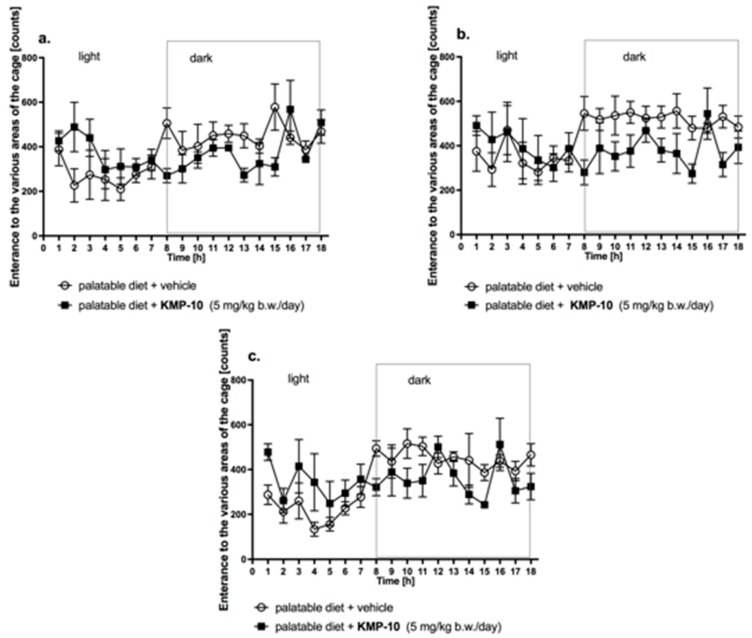
Changes in the spontaneous activity after single (**a**) and fourteen (**b**) and twenty (**c**) day administration of KMP-10 to rats fed palatable diet. Results are mean ± SEM; n = 8, comparisons versus the vehicle-treated excessive eating control groups (two-way ANOVA).

In the group treated with the tested compound and fed the palatable feed, no statistically significant effect on the spontaneous activity was observed in comparison with the control group fed the palatable feed on day 1, 14 or 20 (Figure 16).

#### 2.4.6. Discussion on the Influence of KMP-10 on Metabolism

The 5-HT_6_ receptor blockade has been implicated in the reduction of food intake, body weight, visceral adiposity, and insulin resistance [30]. 5-HT_6_ receptor antagonists block the serotonin-dependent activation of γ-aminobutyric acid (GABA) neurons, which results in a reduction of inhibitory effects of GABA on proopiomelanocortin neurons in the arcuate nucleus, with the subsequent inhibition of hunger signal induction [31]. Our previous results indicated that an administration of KMP-10 to rats in the overeating model caused a significantly lower increase of body mass when compared to that of the control group [19]. The results of the present study suggest a reduced calorie intake by rats as a probable reason. Food consumption in rats and mice can be decreased not only through the enhancement of satiety but also because of various factors including stress, sickness, sedation and drug-induced toxicity [32]. In our studies, the effect of compound KMP-10 on spontaneous activity was not observed. Hence, it is certain that the weight reduction and calorie intake were not associated with sedation. In order to exclude other reasons for the lower food intake and body mass increase, further studies are required. Nevertheless, it is worth underlining that the confirmed significant action of KMP-10 on 5-HT_6_R can be a basis for the inhibition of hunger signal induction caused by this compound. This is consistent to the previous lines of evidence that indicated an anorexic effect of other 5-HT_6_R ligands, and effect which was associated with a reduced food intake via the mechanism related to the enhancement of satiety [30]. Furthermore, comparable results in terms of parameters, such as body weight and the amount of calories consumed, have been provided by earlier studies for another 5-HT_6_R ligand, Idalopirdine [33]. In contrast to KMP-10, Idalopirdine also compensated for elevated glucose levels and significantly reduced the amount of visceral fat. KMP-10 did not reduce the amount of either peritoneal fat or plasma triglyceride and glucose. However, it should be emphasized that the administration of KMP-10 caused a significant increase in the level of HDL-cholesterol in the plasma. Thus, the correction of metabolic changes certainly started in the right direction.

## 3. Materials and Methods

### 3.1. Molecular Modeling

The procedure of 5-HT_6_R homology model generation was based on the β_2_ adrenergic receptor template, and docking studies were performed according to our methods described earlier [17,18,19].

### 3.2. ADME-Tox Parameters In vitro

#### 3.2.1. References

The compounds used as the references—caffeine (CFN), doxorubicin (DX), ketoconazole (KE), nonyl-4-hydroxyquinoline-*N*-oxide (NQNO), norfloxacin (NFX), sulfaphenazole (SE), quinidine (QD) and warfarin (WFN)—were obtained from Sigma-Aldrich (St. Louis, MO, USA). The references for Pgp activity and pharmacokinetic studies—verapamil (VL) and Na_3_VO_4_—were provided with the luminescent Pgp-Glo^TM^ Assay System (Promega, Madison, WI, USA).

#### 3.2.2. Permeability Assay

The PAMPA Plate System Gentest^TM^ was purchased from Corning (Tewksbury, MA, USA). The 10 mM stocks (DMSO) of the examined 5-HT_6_R antagonists were diluted to 200 µM in PBS (phosphate-buffered saline, pH 7.4). Those solutions were added at 300 µL to the donor wells, whereas wells on the receiver plate were filled with 200 µL of PBS. After 5 h of incubation at room temperature, 100 µL of solution were taken from each well and 100 µL of 200 µM of internal standard (IS) were added. Samples prepared in this way were evaluated by the UPLC/MS Waters ACQUITY^TM^ TQD system with the TQ Detector (Waters, Milford, CT, USA) to estimate the concentrations from donor and acceptor wells, as described previously [22]. The assay was performed in triplicate. The permeability value (*Pe*) of examined compounds was calculated using the formula from the literature sources [21]. CFN and NFX were used as references.

#### 3.2.3. Permeability Assay with Using Caco-2

The Caco-2 (ATCC^®^ HTB-37^TM^) cell line was purchased from American Type Culture Collection (ATCC) (Manassas, VG, USA). The cells were cultivated in Dulbecco’s Modified Eagle’s Medium (DMEM) supplemented with 10% fetal bovine serum (FBS) in a humidified atmosphere of 5% CO_2_. The medium was changed every two days, and the cells were subcultured at 70%–80% confluence. The Corning^®^ 3413 Transwell^®^ 6.5 mm polycarbonate membrane inserts with 0.4 µm pores were purchased from Sigma-Aldrich (Saint Louis, MO, USA). The inserts were pretreated first with 50 µL of the medium for two minutes. Next, the 200 µL of cells were seeded at 2 × 10^−4^ concentration per insert in apical compartment, whereas 600 µL was added to the basolateral one. The plate was incubated at 37 °C for 14 h, and the non-adherent cells were removed. TEER (transepithelial electrical resistance) measurements was started from 18 days after seeding by Millicell ERS-2 Volt-Ohm Meter (Merck Millipore, Burlington, MA, USA). The proper monolayer integrity was determined at 20 days after seeding. Then, the monolayer was rinsed with HBSS (Hank’s balanced salt solution) and KMP-10, and the highly permeable reference CFN was added at a 10 µM concentration with the HBSS into the apical chambers. The 600 µL of HBSS was added to the basolateral compartments. Lucifer yellow (5 µM) was also added to the apical chambers as the membrane integrity marker. The plate was placed in the orbital shaker (60 rpm) for 2 h at 37 °C. The compounds’ concentrations in apical and basolateral wells were analyzed using the UPLC-MS method with IS. To confirm the membrane integrity, the fluorescence of lucifer yellow was measured in basolateral compartment by EnSpire multiplate reader (Perkin Elmer, Waltham, MA, USA).

The apparent permeability *P_app_* was calculated from two experiments according to the following formula [34]:

*P_app_*= dc/dt × V/(A × C_0_) (1)

where:

dc/dt—the change in concentration in the receiving compartment over time
V—volume of the solution in the receiving compartment (mL)
A—surface area of the membrane (cm^2^)
C_0_—the initial concentration in the donor compartment (µM).

#### 3.2.4. Affinity to P-glycoprotein

The affinity to P-glycoprotein test was performed as described previously [22,35,36,37] by using commercial the Pgp-Glo^TM^ Assay System (Promega, Madison, WI, USA), accordingly to the manufacturer’s protocol. The reactions were prepared in triplicate in white polystyrene, flat-bottom Nunc^TM^ MicroWell^TM^ 96-well microplates (Thermo Scientific, Waltham, MA USA). The measurements of ATP consuming by Pgp was possible due to the light-generating reaction of firefly luciferase, and these measurments were read by a microplate reader (EnSpire) in luminescence mode. The VL was applied as the reference with a high stimulatory activity on Pgp. KMP-10 and DJ-18 were tested in triplicates at 100 µM.

#### 3.2.5. Plasma Protein Binding

The study was performed with use of the commercial TRANSIL^XL^ PPB Assay (Sovicell, Leipzig, Germany). The test was performed according to recommendations from the protocol provided by the manufacturer. Before the assay, the plate was thawed at room temperature for 3 h. The compounds (stock solution in DMSO) were solved in a PBS buffer up to 320 µM. To each well of the 8-well tube units (six wells containing different concentrations of human serum albumin (HSA) and α_1_-acid glycoprotein (AGP) mixed in physiological ratio of 24:1 and two wells of references), 15 µL of the tested compound solution was added to obtain 5 µM final concentration of WFN and KMP-10. The plate was incubated on a plate shaker at 1000 rpm for 12 min. Next, the plate was centrifugated at 750 g for 10 min. The supernatants were collected and analyzed by LC/MS. The PPB parameters of compound KMP-10 and the highly bound reference WFN were calculated using the following equation:

(2) $$K_{D}=\frac{\left[A\right]\times \left[P\right]}{\left[AP\right]}$$

where:

[A]—free concentration of drug
[P]—free concentration of protein
[AP]—concentration of drug A bound to the protein P.

The total fraction bound was estimated by:

(3) $$f_{b}=1-\frac{1}{1+\frac{\left[\mathrm{HSA}\right]}{K_{D}^{HSA}}+\frac{\left[\mathrm{AGP}\right]}{K_{D}^{AGP}}}$$

#### 3.2.6. Metabolic Stability

The metabolic stability assays were performed as described in our previous articles [22,35,36,37]. The pharmacokinetic parameters of compounds were estimated by using human liver microsomes (HLMs) (Sigma-Aldrich, St. Louis, MO, USA). The *t_1/2_* values and intrinsic clearances (*CL_int_*) were calculated with IS by using the protocols and formulas proposed by Obach [38]. The incubations were terminated at 5, 15, 30 and 45 min. VL was used as the reference unstable drug.

The in vitro evaluation of metabolic pathways was performed by the prolonged, 120 min incubation of compounds with HLMs. The in silico prediction of metabolic biotransformations was performed by MetaSite 6.0.1 software (Molecular Discovery Ltd., Hertfordshire, UK) [39].

#### 3.2.7. Drug–Drug Interaction (DDI)

The study was performed using the CYP3A4 P450-Glo^TM^ and CYP2D6 P450-Glo^TM^ commercial tests provided by Promega (Madison, WI, USA). The influence of compounds on CYPs activity was tested in white polystyrene, flat-bottom Nunc^TM^ MicroWell^TM^ 96-well microplates (Thermo Scientific, Waltham, MA USA), and the bioluminescence signal was measured with a microplate reader (EnSpire) in luminescence mode, as described previously [22,35,36,37]. KMP-10 and DJ-18 were tested in triplicate in the range of 0.01–25 µM for both isoforms’ P450 cytochrome. KE and QD were used as the reference compounds for CYP3A4 and CYP2D6, respectively.

#### 3.2.8. Ames Test

The mutagenic activity was assessed by using the Ames MPF (Microplate Format Mutagenicity) Assay [22,36,37,38] provided by Xenometrix AG (Allschwill, Switzerland), with the use of bacteria *Salmonella typhimurium* strain TA100, thus enabling the detection of base-pair substitution. The growth of reverted bacteria in 384-well plates was observed after 72 h of incubation at 37 °C throughout the change of the color of the indicator medium from purple to yellow. The compounds were examined in triplicate at concentrations 1 and 10 µM. The bacterial medium absorbance was measured with a microplate reader (EnSpire) at the wavelength 420 nm. The mutagenicity was expressed in fold increase above medium control baseline (MCB), where MCB ≥ 2 was considered as the mutagen alert. NQNO was used as reference mutagen.

#### 3.2.9. Hepatotoxicity Assay

Hepatotoxicity was estimated using the hepatoma HepG2 (ATCC^®^ HB-8065^TM^) cell line according to previously described protocols [22,35,36,37]. The CellTiter 96^®^ AQueous Non-Radioactive Cell Proliferation Assay was obtained from Promega (Madison, WI, USA). The compounds were investigated in quadruplicate at four concentrations (0.1–100 µM) for 72h. The antiproliferative drug DX was used as a positive control at a dose of 1 µM.

### 3.3. Behavioral Test In vivo

#### 3.3.1. Animals

The experiments were performed on male Wistar rats (200–220 g) obtained from an accredited animal facility at the Jagiellonian University Medical College, Poland. The animals were housed in group of four in controlled environment (ambient temperature 21 ± 2 °C; relative humidity 50%–60%; 12-h light/dark cycles (lights on at 8:00). Standard laboratory food (LSM-B) and filtered water were freely available. Animals were randomly assigned to treatment groups. All the experiments were performed by two observers unaware of the studied treatment between 9:00 and 14:00 on separate groups of animals. All animals were used only once. Procedures involving animals and their care were conducted in accordance with current European Community and Polish legislation on animal experimentation. Additionally, all efforts were made to minimize animal suffering and to use only the number of animals necessary to produce reliable scientific data. The experimental protocols and procedures described in this manuscript were approved by the I Local Ethics Commission in Cracow (no 200/2018), complied with the European Communities Council Directive of 24 November 1986 (86/609/EEC), and were in accordance with the 1996 National Institutes of Health Guide for the Care and Use of Laboratory Animals.

#### 3.3.2. Drugs

All compounds were suspended in 1% Tween 80 immediately before administration in a volume of 2 mL/kg. Compounds were intraperitoneally administered 60 min before testing. Control animals received vehicle (1% Tween 80) according to the same schedule.

#### 3.3.3. Behavioral Procedures in Rats

##### Elevated Plus-Maze Test (EPM Test)

The testing procedure was based on a method described by [40]. The plus-maze apparatus (an automated device produced by Campden Instruments Ltd. (United Kingdom) was made of durable, high density, non-porous black plastic, elevated to a height of 50 cm, and consisted of two open arms (50 × 10 cm) and two closed arms (50 × 10 cm, and 30 cm high walls) arranged so that the two arms of each type were opposite each other. The floor of the plus-maze was made of infrared transparent material, which means that there were no visible sensors. The plus-maze apparatus was connected to PC software by a control chassis. The experiments were conducted in a darkened room, and only the center of the maze was illuminated with low-intensity light (30 lux measured on the maze level). Each rat was gently placed in the center of the plus-maze, facing one of the closed arms, immediately after a 5-min adaptation period in a plastic black box (60 × 60 × 35 cm), to increase the overall activity in the EPM. During a 5-min test period, an automated motor monitor system recorded the number of entries into the closed and open arms and the time spent in either type of arm. The device counted an effective arm-entry when the four paws of a rat were into any arm. The maze was thoroughly cleaned after each trial. The EPM test is an “unconditional” anxiety-like test based on rodents’ natural aversion to heights and open space.

##### Exploratory Activity Measured in the EPM Test

The experiment was performed using an EPM apparatus (details see above). Total ambulation (the total distance covered by a rat and ambulation along the X and Y axes) was taken into account to discern drug effects on general activity from those on open-arm exploration during a 5-min test period (i.e., the time equal to the observation period in the EPM test). Rats’ behavior was not videotaped during the test.

##### Novel Object Recognition (NOR) Test

Five days before the experiment, the rats were transferred to the laboratory, labeled, and, thereafter, left to acclimate to the new environment. The animals were handled every five days before experiments to minimize the stress reaction. This protocol was adapted from the original work [41,42]. The test session comprising two trials separated by an inter-trial interval (ITI) of 1 h was carried out on the next day. During the first trial (familiarization, T1), two identical objects (A1 and A2) were presented in the opposite corners of the open field, approximately 10 cm from the walls. During the second trial (recognition, T2), one of the A objects was replaced by a novel object B so that the animals were presented with the A = familiar and B = novel objects. Both trials lasted for 3 min, and the animals were returned to their home cages after T1. The objects used were the metal Coca-Cola cans and the glass jars filled with the sand. The heights of the objects were comparable (~12 cm), and the objects were heavy enough to not be displaced by the animals. The sequence of presentations and the location of the objects was randomly assigned to each rat. After each measurement, the floor was cleaned and dried.

The animals explored the objects by looking, licking, sniffing or touching the object but not when leaning against, standing or sitting on the object. Any rat exploring the two objects for less than 5 s within 3 min of T1 or T2 was eliminated from the study. The exploration time of the objects was measured by blind experimenter. Based on exploration time (E) of the two objects during T2, the discrimination index (DI) was calculated according to the formula: DI = (EB − EA)/(EA + EB). Using this metric, scores approaching zero reflected no preference, positive values reflected a preference for the novel object, and negative numbers reflected a preference for the familiar.

Scopolamine and MK-801, used to attenuate learning, were administered at the dose of 0.1 mg/kg (s.c. and i.p., respectively) 30 min before the familiarization phase (T1), while the investigated compounds were given 60 min before T1 session.

#### 3.3.4. Statistical Analysis

The data of behavioral studies were evaluated by an analysis of variance one-way ANOVA followed by Bonferroni’s post-hoc test (statistical significance set at *p* < 0.05).

### 3.4. Assays of Influence on Metabolism In vivo

#### 3.4.1. Animals

The experiments were carried out on male Wistar rats. Their initial body weight was 210–230 g. The animals were housed in pairs in plastic cages in constant temperature facilities exposed to a light–dark cycle; water and food were available ad libitum. The control and experimental groups consisted of eight animals each. All experiments were conducted according to the guidelines of the Animal Use and Care Committee of the Jagiellonian University and were approved for realization (2013 and 2015, Poland; Permissions No 136/2013 and 258/2015).

#### 3.4.2. Drugs

Heparin was delivered by Polfa Warszawa S.A. (Warsaw, Poland), and thiopental sodium was obtained from Sandoz GmbH, (Kundl, Austria).

#### 3.4.3. The Effect of KMP-10 on Food and Water Intake by Non-Obese Rats Fed Palatable Diet (Model of Excessive Eating)

In order to determine the anorectic activity of KMP-10, its effect on caloric and water intake in the model of excessive eating was assessed [43,44]. Male Wistar rats were housed in pairs. Two groups of 8 rats were fed diets consisting of milk chocolate with nuts, cheese, salted peanuts, and 7% condensed milk; these rats also had access to a standard feed (Labofeed B, Morawski Manufacturer Feed, Poland) and water ad libitum for 3 weeks. The palatable control group (palatable diet + vehicle) received vehicle (1% Tween 80, i.p.), while the palatable test group (palatable diet + KMP-10 5 mg) was intraperitoneally injected with KMP-10 at the dose 5 mg/kg b.w./day dissolved in 1% Tween 80. The intakes of food and water were evaluated daily. On the 22th day, 20 min after the i.p. administration of heparin 1000 units/rat and thiopental (70 mg/kg b.w.), blood, peritoneal fat pads, liver were collected from animals.

The palatable diet contained 100 g of peanuts—614 kcal; 100 mL of condensed milk—131 kcal; 100 g of milk chocolate with hazelnuts—195 kcal; and 100 g of cheese (Greek type)—270 kcal.

The standard diet contained 100 g of feed—280 kcal.

The spontaneous activity of rats was measured on the 1st, 14th and 20th day of the treatment with a special RFID-system—TraffiCage (TSE-Systems, Germany) [45,46]. The animals were subcutaneously implanted with transmitter identification (RFID), which enabled the presence and time spent in different areas of the cage to be counted; the data were then grouped in a special computer program.

#### 3.4.4. The Effect of KMP-10 on Food and Water Intake by Non-Obese Rats Fed Only with Standard Diet

Male Wistar rats (190–220 g) were housed in pair. The control group (standard diet + vehicle) received vehicle (1% Tween 80, i.p.), while the test group (standard diet + KMP-10 5 mg) was intraperitoneally injected with KMP-10 at the dose 5 mg/kg b.w./day dissolved in 1% Tween 80. The intakes of food and water were evaluated daily.

#### 3.4.5. Influence of KMP-10 on Glucose, Cholesterol or Triglyceride Levels in Blood

To determine the glucose or cholesterol or triglyceride levels in blood, standard enzymatic and spectrophotometric tests (Biomaxima S.A. Lublin, Poland) were used. The substrate was decomposed with enzymes that were appropriate for the relevant product, which was converted to a colored compound. Coloration was proportional to the concentration. The absorbance was measured at a wavelength of 500 nm.

#### 3.4.6. Statistical Analysis

Statistical calculations were performed using the GraphPad Prism 6 program. Results are given as arithmetic means with a standard error of the mean. Statistical significance was calculated using the Student’s *t*-test (if two groups were compared) or a two-way ANOVA with Sidak’s multiple comparison test post-hoc. Differences were considered statistically significant at: * *p* ≤ 0.05, ** *p* ≤ 0.01, *** *p* ≤ 0.001.

## 4. Conclusions

In response to the scientific and therapeutic challenge of medicinal chemistry to expand the insufficient chemical space of active 5-HT_6_R ligands, this work has explored the most active members of the original, non-indole and non-sulfone, triazine-hydantoin derivatives family. The comprehensive studies performed, including docking, ADMET assays in vitro, and pharmacological tests in vivo have shown beneficial properties of both the 2-naphthyl substituent or the general topology of 3-((4-amino-6-(4-methylpiperazin-1-yl)-1,3,5-triazin-2-yl)methyl)-5-methyl-5-(naphthalen-2-yl)imidazolidine-2,4-dione (KMP-10). These structural properties seem to be responsible for satisfying 5-HT_6_R binding in a different mode from that of indole-containing (Idalopirdine) and sulfone-containing (SUVN-502) antagonists. This mode can be crucial for a contribution in different response in cellular level and is consequently promising for either potential new therapy or for the clarification of the “agonist–antagonist paradox” observed for the ligands of 5-HT_6_R. A predominant role of the naphthyl-containing 5-aryl-5-methyl-hydantoin topology (KMP-10) in comparison to the 5,5-dimethyl-1-arylmethyl-hydantoin one (DJ-18) can be seen not only in the receptor-binding aspect but also in the “druglikeness” properties investigated in vitro. The obtained results indicated more satisfying properties of KMP-10, with respect to DJ-18, in most of the ADMET assays performed. In particular, better pharmacokinetics properties (*CL_int_*, *t_1/2_*) and lower risk of both DDI and toxic effects of KMP-10 were confirmed. Finally, the 5-naphthylhydantoin derivative KMP-10 demonstrated beneficial activities in the primary screening in vivo, including significant anxiolytic-like effects in both “conditional” Vogel- and “unconditional” EPM assays, antidepressive-like properties in the Porsolt model [19] and procognitive action, expressed as the potency to reverse scopolamine-induced memory impairment in rats as well as favorable influence on body mass and metabolism in rats fed with the palatable feed.

Taking the aforementioned data into account, the 5-naphthylhydantoin-1,3,5-triazine derivative KMP-10 seems to be an interesting new lead-structure for either further pharmacological studies or chemical modifications in order to find new therapeutic solutions via 5-HT_6_ receptor regulation.

## Figures and Tables

**Table 1:** Affinities for serotonin/dopamine receptors of hydantoin-triazine compounds DJ-18 and KMP-10 [19].

Cpd	*K_i_* (nM) ^a^				
	5-HT_6_ [^3^H]-LSD	D_2_ [^3^H]-Raclopride	5-HT_1A_ [^3^H]-8-OH-DPAT	5-HT_2A_ [^3^H]-Ketanserin	5-HT_7_ [^3^H]-5-CT
**DJ-18**	127	4098	23,300	nt	3711
**KMP-10**	87	4247	14,160	17,170	514
**Ref.**	[7] ^b^	[9] ^b^	[20] ^c^	-	[18] ^d^

^a^ Tested experimentally in the radioligand binding assay, binding affinity, *K_i_*, expressed as the average of at least two independent experiments; nt—not tested. ^b–d^ Reference ligands for GPCRs investigated: ^b^ olanzapine, ^c^ buspirone, ^d^ clozapine [19].

**Table 2:** Absorption, plasma protein binding (PPB) and pharmacokinetic properties determined in vitro.

Compound	PAMPA *Pe* * [10^−6^ cm/s] ± SD	Caco-2 *P_app_* ** [10^−6^ cm/s] ± SD	PPB *K_D_* *** [μM]	PPB *f_b_* *** [%] ± SD	Half-Life *t_1/2_* **** [min]	Intrinsic Clearance *CL_int_* **** [mL min^−1^ kg^−1^]	
**DJ-18**	24.9 ± 0.45	NT	NT	NT	187.29	4.32	
**KMP-10**	3.76 ± 0.76	6.27 ± 0.30	112	84.5 ± 6.04	238.96	3.74	
**References**	Caffeine 15.1 ± 0.40 Norfloxacin 0.56 ± 0.13	Caffeine 22.04 ± 0.38	Warfarin 9.50	Warfarin 98.5 ± 2.10	Verapamil 30.39	Verapamil 26.76	

NT—not tested, * tested in triplicate, ** data from two experiments, *** calculated according to manufacturer recommendations from eight wells—two references and six wells with increasing immobilized biological phases; **** result based on four independent reactions terminated at different time points.

**Table 3:** The metabolic pathways of DJ-18 and KMP-10.

Substrate	Molecular Mass (*m*/*z*)	Retention Time (min)	Molecular Mass of the Metabolite (*m*/*z*)	Metabolic Pathway
**DJ-18**	459.3	3.99	**M1** 445.34	*demethylation*
		4.21	**M2** 475.38	*hydroxylation*
		4.42	**M3** 461.36	*demethylation and hydroxylation*
		3.40	**M4** 475.38	*hydroxylation*
**KMP-10**	447.27	3.45	**M1** 433.25	*demethylation*
		3.65	**M2** 463.22	*hydroxylation*
		3.82	**M3** 449.26	*demethylation and hydroxylation*
		4.26	**M4** 461.23	*hydroxylation and dehydrogenation*

## Supplementary Materials

The following are available online, Figure S1: MS spectra of compound DJ-18 and its main metabolite M1, Figure S2: MS/MS ion fragment analysis of compound DJ-18 and its main metabolite M1, Figure S3: MS spectra of compound KMP-10 and its main metabolite M1, Figure S4: MS/MS ion fragment analysis of compound DJ-18 and its main metabolite M1.

## References

[1] Monsma F.J., Shen Y., Ward R.P., Hamblin M.W., Sibley D.R. (1993). Cloning and expression of a novel serotonin receptor with high affinity for tricyclic psychotropic drugs. Mol. Pharmacol..

[2] Ruat M., Traiffort E., Arrang J.M., Tardivellacombe J., Diaz J., Leurs R., Schwartz J.C. (1993). A Novel Rat Serotonin (5-HT6) Receptor: Molecular Cloning, Localization and Stimulation of cAMP Accumulation. Biochem. Biophys. Res. Commun..

[3] Yun H.-M., Rhim H. (2011). The Serotonin-6 Receptor as a Novel Therapeutic Target. Exp. Neurobiol..

[4] Karila D., Freret T., Bouet V., Boulouard M., Dallemagne P., Rochais C. (2015). Therapeutic Potential of 5-HT 6 Receptor Agonists. J. Med. Chem..

[5] Leiser S.C., Li Y., Pehrson A.L., Dale E., Smagin G., Sanchez C. (2015). Serotonergic Regulation of Prefrontal Cortical Circuitries Involved in Cognitive Processing: A Review of Individual 5-HT Receptor Mechanisms and Concerted Effects of 5-HT Receptors Exemplified by the Multimodal Antidepressant Vortioxetine. ACS Chem. Neurosci..

[6] Nikiforuk A. (2014). The procognitive effects of 5-HT6 receptor ligands in animal models of schizophrenia. Rev. Neurosci..

[7] Benhamú B., Martín-Fontecha M., Vázquez-Villa H., Pardo L., López-Rodríguez M.L. (2014). Serotonin 5-HT 6 Receptor Antagonists for the Treatment of Cognitive Deficiency in Alzheimer’s Disease. J. Med. Chem..

[8] de Jong I.E.M., Mørk A. (2017). Antagonism of the 5-HT6 receptor—Preclinical rationale for the treatment of Alzheimer’s disease. Neuropharmacology.

[9] Kenakin T. (2009). Biased agonism. F1000 Biol. Rep..

[10] Ivanenkov Y.A., Majouga A.G., Veselov M.S., Chufarova N.V., Baranovsky S.S., Filkov G. (2014). Computational approaches to the design of novel 5-HT6R ligands. Rev. Neurosci..

[11] Grychowska K., Satała G., Kos T., Partyka A., Colacino E., Chaumont-Dubel S., Bantreil X., Wesołowska A., Pawłowski M., Martinez J. (2016). Novel 1H-Pyrrolo[3,2-c]quinoline Based 5-HT6 Receptor Antagonists with Potential Application for the Treatment of Cognitive Disorders Associated with Alzheimer’s Disease. ACS Chem. Neurosci..

[12] Zajdel P., Marciniec K., Satała G., Canale V., Kos T., Partyka A., Jastrzębska-Więsek M., Wesołowska A., Basińska-Ziobroń A., Wójcikowski J. (2016). N1-Azinylsulfonyl-1H-indoles: 5-HT6 Receptor Antagonists with Procognitive and Antidepressant-Like Properties. ACS Med. Chem. Lett..

[13] Kelemen Á., Satała G., Bojarski A., Keserű G. (2017). Spiro[pyrrolidine-3,3′-oxindoles] and Their Indoline Analogues as New 5-HT6 Receptor Chemotypes. Molecules.

[14] Staroń J., Mordalski S., Warszycki D., Satała G., Hogendorf A., Bojarski A.J. (2017). Pyrano[2,3,4-cd]indole as a Scaffold for Selective Nonbasic 5-HT6R Ligands. ACS Med. Chem. Lett..

[15] Nirogi R., Abraham R., Benade V., Medapati R.B., Jayarajan P., Bhyrapuneni G., Muddana N., Mekala V.R., Subramanian R., Shinde A. (2019). SUVN-502, a novel, potent, pure, and orally active 5-HT6 receptor antagonist: Pharmacological, behavioral, and neurochemical characterization. Behav. Pharmacol..

[16] Liu K.G., Robichaud A.J. (2009). 5-HT6 antagonists as potential treatment for cognitive dysfunction. Drug Dev. Res..

[17] Łażewska D., Kurczab R., Więcek M., Kamińska K., Satała G., Jastrzębska-Więsek M., Partyka A., Bojarski A.J., Wesołowska A., Kieć-Kononowicz K. (2017). The computer-aided discovery of novel family of the 5-HT6 serotonin receptor ligands among derivatives of 4-benzyl-1,3,5-triazine. Eur. J. Med. Chem..

[18] Łażewska D., Kurczab R., Więcek M., Satała G., Kieć-Kononowicz K., Handzlik J. (2019). Synthesis and computer-aided analysis of the role of linker for novel ligands of the 5-HT6 serotonin receptor among substituted 1,3,5-triazinylpiperazines. Bioorg. Chem..

[19] Kurczab R., Ali W., Łażewska D., Kotańska M., Jastrzębska M., Satała G., Więcek M., Lubelska A., Wesołowska A., Latacz G. (2018). Computer-Aided Studies for Novel Arylhydantoin 1,3,5-Triazine Derivatives as 5-HT6 Serotonin Receptor Ligands with Antidepressive-Like, Anxiolytic and Antiobesity Action In vivo. Molecules.

[20] Hogendorf A.S., Hogendorf A., Kurczab R., Kalinowska-Tłuścik J., Popik P., Nikiforuk A., Krawczyk M., Satała G., Lenda T., Knutelska J. (2019). 2-Aminoimidazole-based antagonists of the 5-HT6 receptor—A new concept in aminergic GPCR ligand design. Eur. J. Med. Chem..

[21] Chen X., Murawski A., Patel K., Crespi C.L., Balimane P.V. (2008). A novel design of artificial membrane for improving the PAMPA model. Pharm. Res..

[22] Latacz G., Lubelska A., Jastrzębska-Więsek M., Partyka A., Marć M.A., Satała G., Wilczyńska D., Kotańska M., Więcek M., Kamińska K. (2019). The 1,3,5-Triazine Derivatives as Innovative Chemical Family of 5-HT6 Serotonin Receptor Agents with Therapeutic Perspectives for Cognitive Impairment. Int. J. Mol. Sci..

[23] Kerns E., Di L. (2008). Drug-Like Properties: Concept, Structure Design and Methods, From ADME to Toxicity Optimization.

[24] Smetanova L., Stetinova V., Kholova D., Kvetina J., Smetana J., Svoboda Z. (2009). Caco-2 cells and Biopharmaceutics Classification System (BCS) for prediction of transepithelial transport of xenobiotics (model drug: Caffeine). Neuro Endocrinol. Lett..

[25] Mullokandov E., Ahn J., Szalkiewicz A. (2014). Protein Binding Drug-Drug Interaction between Warfarin and Tizoxanide in Human Plasma. Austin J. Pharmacol. Ther..

[26] Rosengren A.M., Karlsson B.C.G., Nicholls I.A. (2012). Monitoring the distribution of warfarin in blood plasma. ACS Med. Chem. Lett..

[27] Popiołek-Barczyk K., Łażewska D., Latacz G., Olejarz A., Makuch W., Stark H., Kieć-Kononowicz K., Mika J. (2018). Antinociceptive effects of novel histamine H3 and H4 receptor antagonists and their influence on morphine analgesia of neuropathic pain in the mouse. Br. J. Pharmacol..

[28] Nassar A.F. (2009). Drug Metabolism Handbook: Concepts and Applications.

[29] Nirogi R., Shinde A., Kambhampati R.S., Mohammed A.R., Saraf S.K., Badange R.K., Bandyala T.R., Bhatta V., Bojja K., Reballi V. (2017). Discovery and Development of 1-[(2-Bromophenyl)sulfonyl]-5-methoxy-3-[(4-methyl-1-piperazinyl)methyl]-1H-indole Dimesylate Monohydrate (SUVN-502): A Novel, Potent, Selective and Orally Active Serotonin 6 (5-HT6) Receptor Antagonist for Potential Treatment of Alzheimer’s Disease. J. Med. Chem..

[30] Heal D.J., Smith S.L., Fisas A., Codony X., Buschmann H. (2008). Selective 5-HT_6_ receptor ligands: Progress in the development of a novel pharmacological approach to the treatment of obesity and related metabolic disorders. Pharmacol. Ther..

[31] Sargent B.J., Henderson A.J. (2011). Targeting 5-HT receptors for the treatment of obesity. Curr. Opin. Pharmacol..

[32] Vickers S.P., Jackson H.C., Cheetham S.C. (2011). The utility of animal models to evaluate novel anti-obesity agents. Br. J. Pharmacol..

[33] Kotańska M., Lustyk K., Bucki A., Marcinkowska M., Śniecikowska J., Kołaczkowski M. (2018). Idalopirdine, a selective 5-HT_6_ receptor antagonist, reduces food intake and body weight in a model of excessive eating. Metab Brain Dis..

[34] Vaidyanathan J.B., Walle T. (2001). Transport and metabolism of the tea flavonoid (−)-epicatechin by the human intestinal cell line Caco-2. Pharm. Res..

[35] Latacz G., Hogendorf A.S., Hogendorf A., Lubelska A., Wierońska J.M., Woźniak M., Cieślik P., Kieć-Kononowicz K., Handzlik J., Bojarski A.J. (2018). Search for a 5-CT alternative. In vitro and in vivo evaluation of novel pharmacological tools: 3-(1-alkyl-1H-imidazol-5-yl)-1H-indole-5-carboxamides, low-basicity 5-HT_7_ receptor agonists. Medchemcomm.

[36] Latacz G., Lubelska A., Jastrzębska-Więsek M., Partyka A., Kucwaj-Brysz K., Wesołowska A., Kieć-Kononowicz K., Handzlik J. (2018). MF-8, a novel promising arylpiperazine-hydantoin based 5-HT7 receptor antagonist: In vitro drug-likeness studies and in vivo pharmacological evaluation. Bioorg. Med. Chem. Lett..

[37] Latacz G., Lubelska A., Jastrzębska-Więsek M., Partyka A., Sobiło A., Olejarz A., Kucwaj-Brysz K., Satała G., Bojarski A.J., Wesołowska A. (2017). In the search for a lead structure among series of potent and selective hydantoin 5-HT7R agents: The drug-likeness in vitro study. Chem. Biol. Drug Des..

[38] Obach R.S. (1999). Prediction of human clearance of twenty-nine drugs from hepatic microsomal intrinsic clearance data: An examination of in vitro half-life approach and nonspecific binding to microsomes. Drug Metab. Dispos..

[39] Cruciani G., Carosati E., De Boeck B., Ethirajulu K., Mackie C., Howe T., Vianello R. (2005). MetaSite: Understanding metabolism in human cytochromes from the perspective of the chemist. J. Med. Chem..

[40] Pellow S., File S.E. (1986). Anxiolytic and anxiogenic drug effects on exploratory activity in an elevated plus-maze: A novel test of anxiety in the rat. Pharmacol. Biochem. Behav..

[41] Ennaceur A., Delacour J. (1988). A new one-trial test for neurobiological studies of memory in rats. 1: Behavioral data. Behav. Brain Res..

[42] Zajdel P., Kos T., Marciniec K., Satała G., Canale V., Kamiński K., Hołuj M., Lenda T., Koralewski R., Bednarski M. (2018). Novel multi-target azinesulfonamides of cyclic amine derivatives as potential antipsychotics with pro-social and pro-cognitive effects. Eur. J. Med. Chem..

[43] Kotańska M., Śniecikowska J., Jastrzębska-Więsek M., Kołaczkowski M., Pytka K. (2017). Metabolic and Cardiovascular Benefits and Risks of EMD386088—A 5-HT6 Receptor Partial Agonist and Dopamine Transporter Inhibitor. Front. Neurosci..

[44] Kotańska M., Kulig K., Marcinkowska M., Bednarski M., Malawska K., Zaręba P. (2018). Metabolic benefits of 1-(3-(4-(o-tolyl)piperazin-1-yl)propyl)pyrrolidin-2-one: A non-selective α-adrenoceptor antagonist. J. Endocrinol. Investig..

[45] Dudek M., Marcinkowska M., Bucki A., Olczyk A., Kołaczkowski M. (2015). Idalopirdine—A small molecule antagonist of 5-HT6 with therapeutic potential against obesity. Metab. Brain Dis..

[46] Dudek M., Knutelska J., Bednarski M., Nowiński L., Zygmunt M., Kazek G., Mordyl B., Głuch-Lutwin M., Zaręba P., Kulig K. (2016). Pyrrolidin-2-one derivatives may reduce body weight in rats with diet-induced obesity. Eur. J. Pharmacol..

