# Supplementary material for: Are the Hydantoin-1,3,5-triazine 5-HT6R Ligands a Hope to a Find New Procognitive and Anti-Obesity Drug? Considerations Based on Primary In Vivo Assays and ADME-Tox Profile In Vitro
Source: Molecules. 2019 Dec 6;24(24):4472. doi: 10.3390/molecules24244472 (PMC6943527; doi:10.3390/molecules24244472)
Supplement: Supplementary file 1 [file molecules-24-04472-s001.pdf]

Supporting Information

Are the hydantoin-1,3,5-triazine 5-HT$_6$R ligands a hope to find new procognitive and anti-obesity drug? Considerations based on primary *in vivo* assays and ADME-Tox profile *in vitro*.

Annamaria Lubelska, Gniewomir Latacz, Magdalena Jastrzębska-Więsek, Magdalena Kotańska, Rafał Kurczab, Anna Partyka, Małgorzata Anna Marć, Daria Wilczyńska, Agata Doroz-Płonka, Dorota Łażewska, Anna Wesołowska, Katarzyna Kieć-Kononowicz and Jadwiga Handzlik*

I. Characteristics of tested triazines [19]

Purity and chemical characteristics of DJ-18 [19]:

Chemical nomenclature:
1-(4-Chlorobenzyl)-3-((4-amino-6-(4-methylpiperazin-1-yl)-1,3,5-triazin-2-yl)methyl)-5,5-dimethylimidazolidine-
2,4-dione

Chemical formula: $C_{21}H_{27}N_8O_2Cl$ (MW458.95)

Chemical form: White solid, m.p. 204-205 ^{0}C.
NMR analyses:
^{1}H-NMR (300MHz, DMSO-d$_6$) δ [ppm]: 7.38 (s, 4H, Ph-2,3,5,6-H), 6.87 (br. s., 2H, NH2), 4.51 (s, 2H, Ph-CH2), 4.27 (s, 2H, TR-CH2), 3.55 (br. s., 4H, Pp-2,6-H), 2.15 (s, 7H, Pp-3,5-H +Pp-CH3), 1.26 (s, 6H, 2 CH3).

Purity:
LC/MS+: Purity: 100%, tR = 3.94, (ESI) m/z [M+ H]+ 459.23.

Purity and chemical characteristics of KMP-10:

Chemical nomenclature:
3-((4-Amino-6-(4-methylpiperazin-1-yl)-1,3,5-triazin-2-yl)methyl)-5-methyl-5-(naphthalen-2-yl)imidazolidine-2,4-dione

Chemical formula: $C_{23}H_{26}N_8O_2$ (MW446.50)

Chemical form: White solid, m.p. 144 ^{0}C.

NMR analyses:

^{1}H-NMR (300MHz, DMSO-d_6) δ [ppm]: 9.12 (s, 1H, N1–H), 8.07 (s, 1H, Napht-1-H), 7.93–7.99 (m, 3H,
Napht-4,5,8-H), 7.72–7.74 (d def., 1H, Napht-3-H), 7.54–7.57 (m, 2H, Napht-6,7-H), 6.85 (s, 2H, NH_2), 4.27 (s, 2H, N3–CH_2), 3.40–3.60 (m, 2H, Pp-2,6-H_b), 3.05–3.20 (m, 2H, Pp-2,6-H_a), 2.09 (s, 4H, Pp-3,5-H), 2.00 (s, 3H, N–CH_3), 1.83 (s, 3H, 5-CH_3).

^{13}C-NMR (75MHz, DMSO-d_6) δ [ppm]: 175.56, 172.01, 166.95, 164.53, 155.92, 137.75, 132.96, 132.81, 128.62, 128.57, 127.89, 126.95, 126.90, 124.51, 124.33, 63.56, 53.90, 46.00, 42.56, 42.47, 25.94.

Purity:

LC/MS+: Purity: 99.11%, tR = 3.49, (ESI) m/z [M]+ 447.20.

[19] Kurczab, R.; Ali, W.; Łażewska, D.; Kotańska, M.; Jastrzębska, M.; Satała, G.; Więcek, M.; Lubelska, A.; Wesołowska, A.; Latacz, G.; et al. Computer-Aided Studies for Novel Arylhydantoin 1,3,5-Triazine Derivatives as 5-HT6 Serotonin Receptor Ligands with Antidepressive-Like, Anxiolytic and Antiobesity Action In Vivo. *Molecules* **2018**, *23*, 1–26.

II. Additional data for *in vivo* assays performed

Table S1. Effect of KMP-10 on total exploration in the EPM test in rats.

Treatment	Dose (mg/kg)	Total distance (cm)	X Ambulation	Y Ambulation
**Vehicle**	0	3375 ± 344	120 ± 15	89 ± 10
**KMP-10**	0.3	3240 ± 209	116 ± 18	98 ± 20
	1	4558 ± 205	152 ± 17	63 ± 12
	3	3881 ± 298 F(3,22)=2.192 NS	131 ± 18 F(3,22)=0.883 NS	133 ± 25 F(3,22)=2.708 NS

KMP-10 was given i.p. 60 min before the test. Values represent the mean ± SEM of the total distance, X ambulation, and Y ambulation during 5-min test session compared to the vehicle group (one-way ANOVA followed by Bonferroni's post hoc test); NS=non-significant. N=6-8

II. Additional data for metabolite studies *in vitro*

MS spectra and MS ion fragment analyses of **DJ-18** and its main metabolite

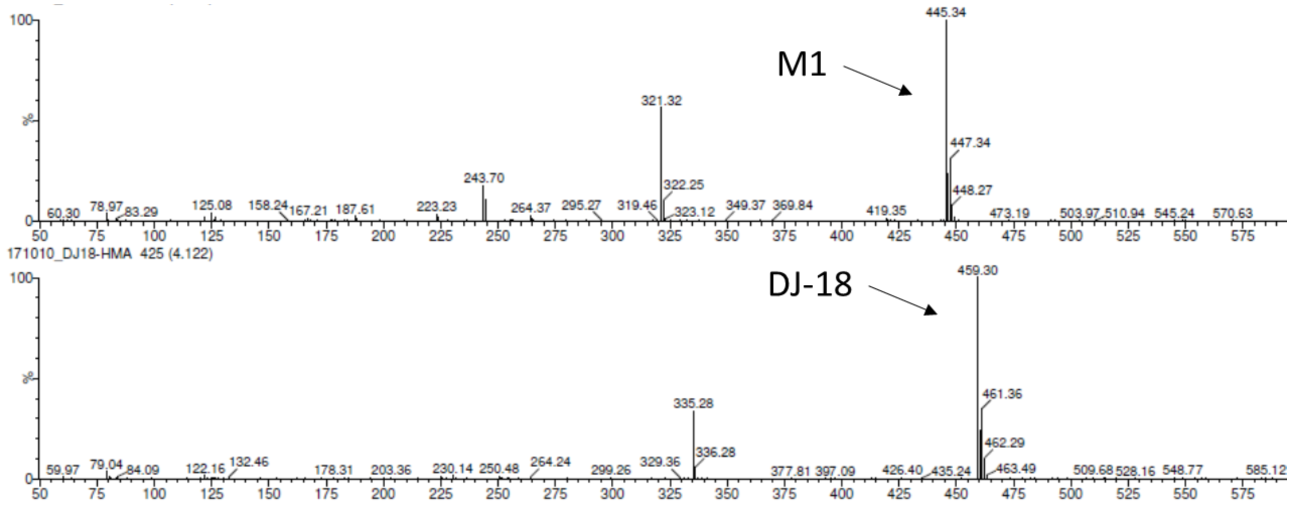

Figure S1 MS spectra of compound **DJ-18** and its main metabolite M1.

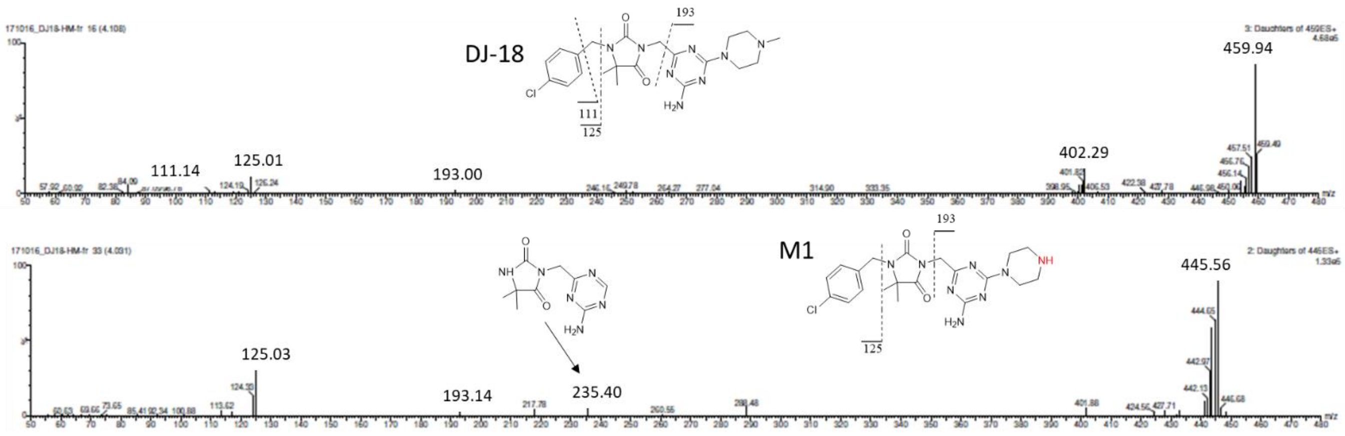

Figure S2 MS/MS ion fragment analysis of compound **DJ-18** and its main metabolite M1.

MS spectra and MS ion fragment analyses of **KMP-10** and its main metabolite

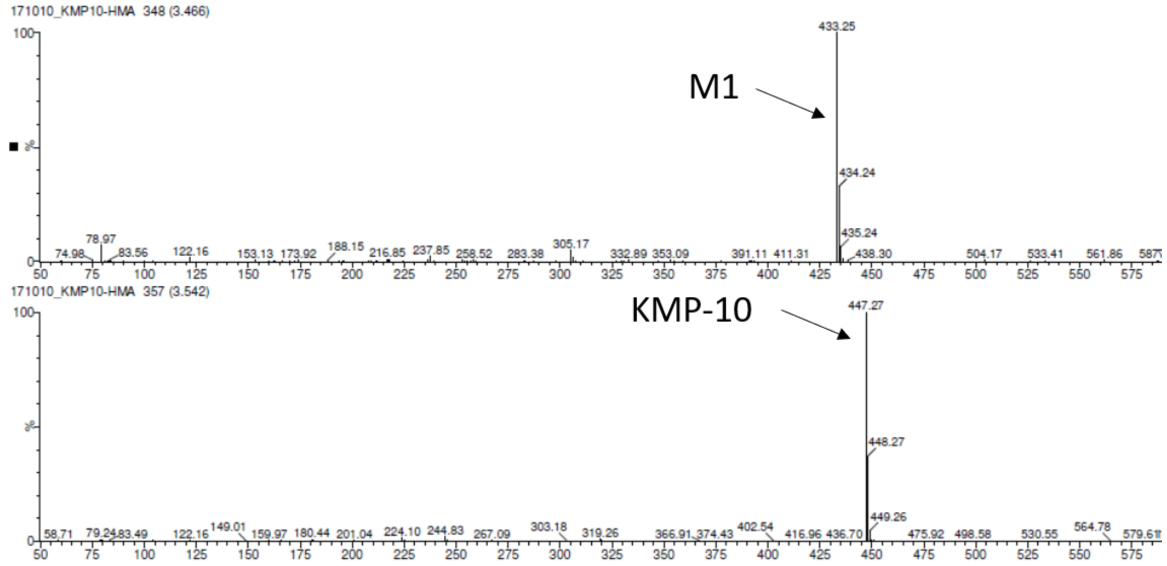

Figure S3 MS spectra of compound **KMP-10** and its main metabolite M1.

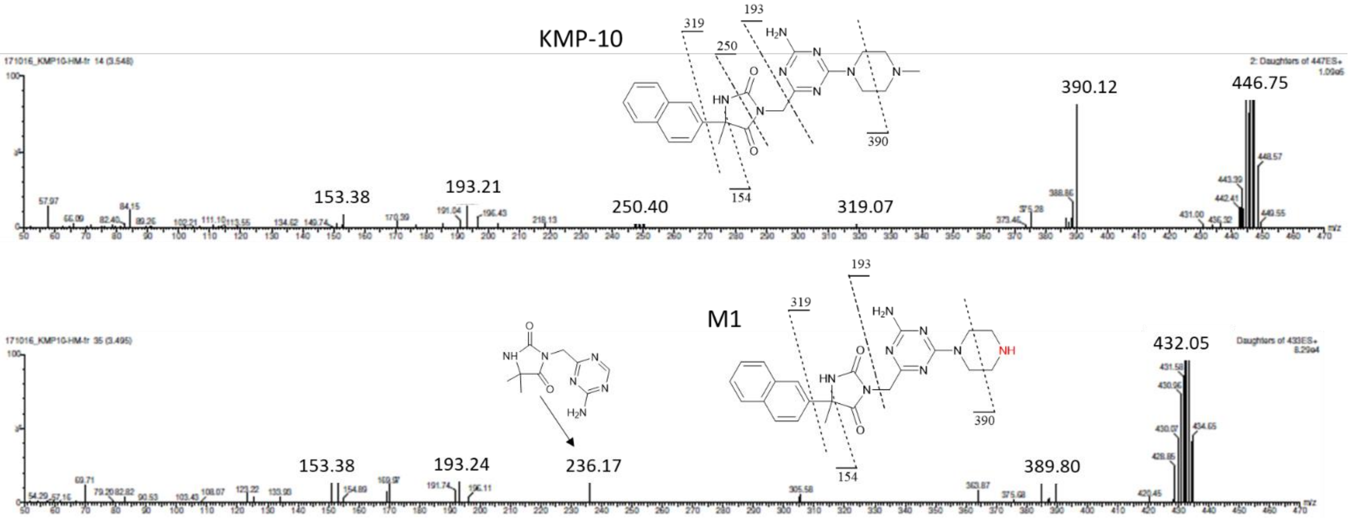

Figure S4 MS/MS ion fragment analysis of compound **DJ-18** and its main metabolite M1.
